# Type II restriction endonuclease R.Hpy188I belongs to the GIY-YIG nuclease superfamily, but exhibits an unusual active site

**DOI:** 10.1186/1472-6807-8-48

**Published:** 2008-11-14

**Authors:** Katarzyna H Kaminska, Mikihiko Kawai, Michal Boniecki, Ichizo Kobayashi, Janusz M Bujnicki

**Affiliations:** 1 Laboratory of Bioinformatics and Protein Engineering, International Institute of Molecular and Cell Biology in Warsaw, Trojdena 4, 02-109 Warsaw, Poland; 2 Laboratory of Bioinformatics, Institute of Molecular Biology and Biotechnology, Faculty of Biology, Adam Mickiewicz University, Umultowska 89, 61-614 Poznan, Poland; 3 Department of Medical Genome Sciences, Graduate School of Frontier Science, The University of Tokyo, 4-6-1 Shirokanedai, Minato-ku, Tokyo 108-8639, Japan; 4 Institute of Medical Science, The University of Tokyo, 4-6-1 Shirokanedai, Minato-ku, Tokyo 108-8639, Japan

## Abstract

**Background:**

Catalytic domains of Type II restriction endonucleases (REases) belong to a few unrelated three-dimensional folds. While the PD-(D/E)XK fold is most common among these enzymes, crystal structures have been also determined for single representatives of two other folds: PLD (R.BfiI) and half-pipe (R.PabI). Bioinformatics analyses supported by mutagenesis experiments suggested that some REases belong to the HNH fold (e.g. R.KpnI), and that a small group represented by R.Eco29kI belongs to the GIY-YIG fold. However, for a large fraction of REases with known sequences, the three-dimensional fold and the architecture of the active site remain unknown, mostly due to extreme sequence divergence that hampers detection of homology to enzymes with known folds.

**Results:**

R.Hpy188I is a Type II REase with unknown structure. PSI-BLAST searches of the non-redundant protein sequence database reveal only 1 homolog (R.HpyF17I, with nearly identical amino acid sequence and the same DNA sequence specificity). Standard application of state-of-the-art protein fold-recognition methods failed to predict the relationship of R.Hpy188I to proteins with known structure or to other protein families. In order to increase the amount of evolutionary information in the multiple sequence alignment, we have expanded our sequence database searches to include sequences from metagenomics projects. This search resulted in identification of 23 further members of R.Hpy188I family, both from metagenomics and the non-redundant database. Moreover, fold-recognition analysis of the extended R.Hpy188I family revealed its relationship to the GIY-YIG domain and allowed for computational modeling of the R.Hpy188I structure. Analysis of the R.Hpy188I model in the light of sequence conservation among its homologs revealed an unusual variant of the active site, in which the typical Tyr residue of the YIG half-motif had been substituted by a Lys residue. Moreover, some of its homologs have the otherwise invariant Arg residue in a non-homologous position in sequence that nonetheless allows for spatial conservation of the guanidino group potentially involved in phosphate binding.

**Conclusion:**

The present study eliminates a significant "white spot" on the structural map of REases. It also provides important insight into sequence-structure-function relationships in the GIY-YIG nuclease superfamily. Our results reveal that in the case of proteins with no or few detectable homologs in the standard "non-redundant" database, it is useful to expand this database by adding the metagenomic sequences, which may provide evolutionary linkage to detect more remote homologs.

## Background

Type II restriction endonucleases (REases) form one of the largest groups of biochemically characterized enzymes (reviews: [1,2]). They usually recognize a short (4–8 bp) palindromic sequence of double-stranded DNA and catalyze the hydrolysis of phosphodiester bonds at precise positions within or close to this sequence, leaving "blunt" ends or "sticky" (5' or 3') overhangs. They form restriction-modification (RM) systems together with DNA methyltransferases (MTases) of the same or a similar sequence specificity, whose enzymatic activity leads to methylation of the target sequence and, consequently, its protection against the cleavage by the REase [3]. Type II RM systems behave as selfish "toxin-antitoxin" genetic modules; they undergo rampant horizontal transfer and parasitize the cells of prokaryotic hosts to ensure the maintenance of their DNA [4-6]. The activity of the RM systems manifests itself by destruction of DNA molecules without the required methylation patterns, e.g. DNA molecules of invading phages or plasmids, or the genomic DNA of their host cells that once had the RM genes but have lost them.

The activity of REases is the target of selection pressure involving various agents: their host, the invading DNA molecules, and their competitors including other RM systems [7-10]. Presumably because of the absence of simple constant selection pressure on the REase activity, they undergo rapid divergence, and as a consequence, different REase families exhibit very little sequence similarity (review: [11]). Besides, there is formidable evidence, mainly from crystallographic analyses, that these enzymes have originated independently in the evolution on at least several occasions.

Thus far, REases have been found to belong to at least five unrelated structural folds. Most of REases belong to the PD-(D/E)XK superfamily of Mg^2+^-dependent nucleases, which also includes various proteins involved in DNA recombination and repair [12,13]. Two REases with different folds have been found to be Mg^2+^-independent: R.BfiI belongs to the phospholipase D (PLD) superfamily of phosphodiesterases [14,15], while R.PabI exhibits a novel "half-pipe" fold [16,17]. A number of REases have been predicted to be related to the HNH superfamily of metal-dependent nucleases, which groups together enzymes with various activities, such as recombinases, DNA repair enzymes, and homing endonucleases [12,18]. For some of these REases from the HNH superfamily, bioinformatics predictions of the active site have been substantiated by mutagenesis; examples include R.KpnI [19], R.MnlI [20], and R.Eco31I [21]. Finally, R.Eco29kI and its two close homologs have been predicted to belong to the GIY-YIG superfamily of nucleases that includes e.g. DNA repair enzymes and homing nucleases [22]; this prediction has been recently supported by mutagenesis of the R.Eco29kI active site [23]. Among of all REase folds, the mechanism of action of GIY-YIG and half-pipe nucleases is least well understood, and no co-crystal structures are available for any member of these superfamilies.

A recent large-scale bioinformatics survey of Type II REase sequences [24] indicated that for about 81% of experimentally characterized (i.e. not putative) enzymes, the three-dimensional fold can be predicted based on advanced bioinformatics analyses, mainly protein fold-recognition and analysis of amino acid conservation patterns and secondary structure prediction (review of methodology: [25]). However, the other REases remain unassigned to known folds and the architectures of their active sites and potential mechanisms of action remain obscure.

R.Hpy188I is one of the REases, for which no fold prediction have been made thus far. R.Hpy188I recognizes the unique sequence, TCNGA, and cleaves the DNA between nucleotides N and G in its recognition sequence to generate a one-base 3' overhang [26]. Its orthologs are found among many, but not all, strains of *Helicobacter pylori *that have been tested with respect to the REase activity [27]. In this work, we present the results of a bioinformatics analysis that has detected remote relationship between R.Hpy188I and known GIY-YIG nucleases thanks to utilization of metagenomics sequences to generate a multiple sequence alignment with enhanced evolutionary information. We suggest that this approach could be applied to predict structure of other proteins, for which fold-recognition analyses done with standard alignments have failed.

## Results

### Initial bioinformatics analysis of R.Hpy188I and its homologs

The lack of overall sequence conservation among REases, the absence of invariable residues even in the active site and the presence of several alternative folds makes structure prediction and generation of multiple sequence alignments for these enzymes a non-trivial task. In order to predict the structure of R.Hpy188I, we used the GeneSilico meta-server, which is a gateway to a number of third-party algorithms (see Methods). In particular, we predicted the secondary structure of this enzyme and carried out the fold recognition analysis to identify the structures of potentially homologous proteins in the Protein Data Bank that could serve as modeling templates. Unfortunately, querying the meta-server with R.Hpy188I sequence alone has not revealed any significant matches to proteins of known structure (for a discussion of significance thresholds of individual FR methods, see the Methods section). Of all methods used, only HHSEARCH revealed a match to GIY-YIG nucleases, albeit at the 9^th ^position of the ranking, with a score that did not indicate statistical significance (probability 0.113, e-value 68).

Most of fold recognition methods make their predictions not for a single sequence, but for a multiple sequence alignment generated by PSI-BLAST searches of the non-redundant (nr) NCBI database (or of a subset of sequences culled from this database). Analysis of an independently carried out PSI-BLAST run against that database (with e-value threshold of 1e-3) revealed only one nearly identical sequence, of REase R.HpyF17I that exhibits only 1 amino acid difference and 4 additional amino acids at the N terminus (Sapranauskas, R., Lubys, A. and Janulaitis, A. unpublished reference "Cloning and analysis of the TCNGA-specific restriction-modification system from *Helicobacter pylori *strain A17-2"). The results of fold recognition analysis starting from R.HpyF17I or from an alignment of R.Hpy188I and R.HpyF17I, were the same as those starting from R.Hpy188I alone. Thus, R.Hpy188I can be considered an "ORFan" [28], at least with respect to the nr database.

Previously, in the course of bioinformatics analysis of R.NlaIV enzyme, we found that inclusion of sequences from metagenomics projects can increase information content of a multiple sequence alignment and improve detection of remote homologies, in particular for proteins with very few homologs in the nr database [29]. Thus, we carried out a new PSI-BLAST search for R.Hpy188I (also with e-value threshold of 1e-3), of the env_nr database (protein sequences deduced from environmental DNA samples), obtained from the NCBI server database. This search revealed 9 sequences, with e-values ranging from 3e-10 to 3e-4. Again, running FR analyses for these sequences gave no significant matches to any structure. Nonetheless, a PSI-BLAST search of a database comprising both nr AND env_nr revealed an increased number of sequences. In the search, 25 sequences including 18 from marine metagenome [30] were found to exhibit significant scores (e-values < e-4) and a conserved pattern of residues (I/V)-Y-X_9_-(K)-I-G (where X indicates any amino acid residue) associated with a predicted β-hairpin structure that remotely resembled the genuine bipartite GIY-YIG motif. FR analysis of a multiple sequence alignment calculated for the sequences returned by the PSI-BLAST search revealed the relationship of these sequences to the GIY-YIG superfamily, according to the following servers: HHSEARCH (probability 0.946), FFAS (score -12.6), mGenTHREADER (probability 0.422), FUGUE (score 10.3), INUB (score 44.1). According to the Livebench evaluation, all these scores indicate higher reliability than the threshold of approximately 5% false positives (see Methods) and in our experience can be taken as reasonably confident 3D fold prediction. Further, the consensus predictor PCONS selected 1yd0 as a preferred template with score 0.665, a value almost exactly at the threshold. Thus, we estimate that a probability of incorrect fold prediction for the R.Hpy188I family is around 5%.

We conclude that utilization of evolutionary information from metagenomics sequences can greatly increase the information content of a multiple sequence alignment, to the point where a reasonably sized family can be detected for a sequence, which appears as an "ORFan" when only the nr database is considered. An extended multiple sequence alignment that includes metagenomics sequences together with proteins from the nr database can then be used as a sensitive probe in protein fold-recognition, for detection of remote homologies to proteins of known structure.

### Molecular modeling of R.Hpy188I

**Figure 1:**
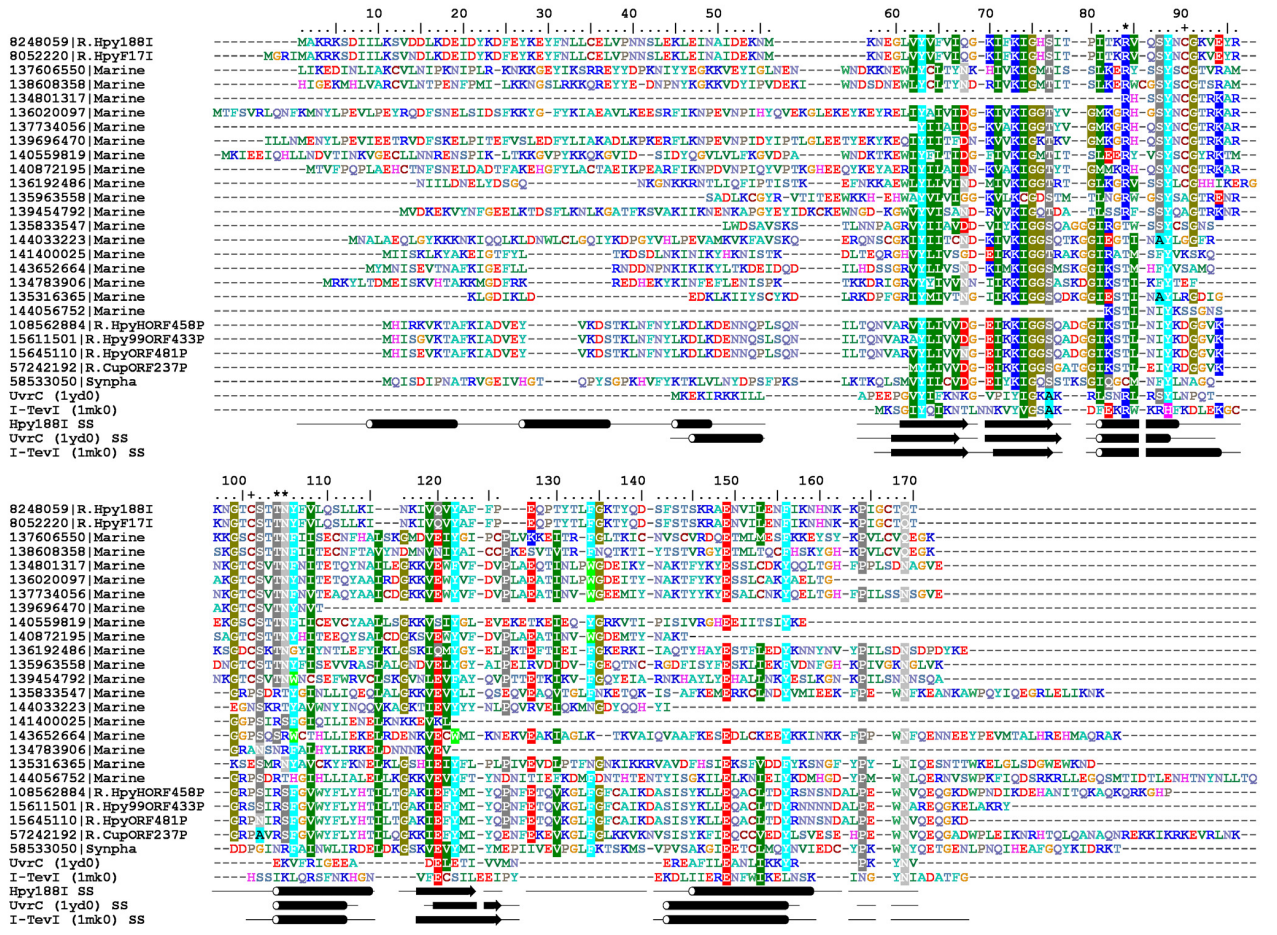
**Multiple sequence alignment of 25 members of the R.Hpy188I family, together with the structurally characterized homologs from the GIY-YIG superfamily, UvrC (1yd0) and I-TevI (1mk0).** The members are indicated by the NCBI GI number followed by the original REBASE name, e.g. R.Hpy188I (for sequences available in REBASE, e.g. R.Hpy188I) or the abbreviated genus and species name with exception of Marine for the marine metagenome and Synpha for *Synechococcus *phage S-PM2. Amino acid residues are colored according to the similarity of their physico-chemical properties. Secondary structure (ss), as determined experimentally for UvrC and I-TevI and predicted for R.Hpy188I (taken from the final model presented in the present study), is indicated below the alignment as tubes (helices) and arrows (strands). The alternative positions of Arg residue (84 or 104 or 105 in R.Hpy188I sequence) are indicated by asterisks (*) above the alignment, whereas the position of the two Cys residues C90 and C101 is indicated by plus characters (+).

It is well known that fold recognition methods can produce artifacts. For instance, sequence alignments to wrong templates can reveal misleading local similarity of amino acid residues, and generate structures that are completely misfolded. Thus, in order to substantiate the sequence-based prediction of membership of R.Hpy188I in the GIY-YIG superfamily (with the confidence of FR predictions estimated to be around 95%), we decided to build a model of its structure and evaluate its quality on the three-dimensional level. Although the GIY-YIG domain of UvrC [31] has been identified as the preferred structure, fold recognition alignments reported by different methods exhibited differences. Thus, we used the "FRankenstein's Monster" approach to simultaneously generate a model of the protein core and optimize the target-template alignment by generation, evaluation, and recombination of alternative models [32,33]. This approach has been evaluated as one of the best template-modeling methods in CASP5 and CASP6; we have also used it to generate accurate models of REases R.SfiI [34] and R.MvaI [35], which were confirmed by independent crystallographic analyses [36,37]. The final alignment (Figure 1) indicated that regions 1–59, 89–103, and 113–121 of R.Hpy188I lack the counterpart in GIY-YIG domains of known structure and cannot be modeled "by homology".

**Figure 2:**
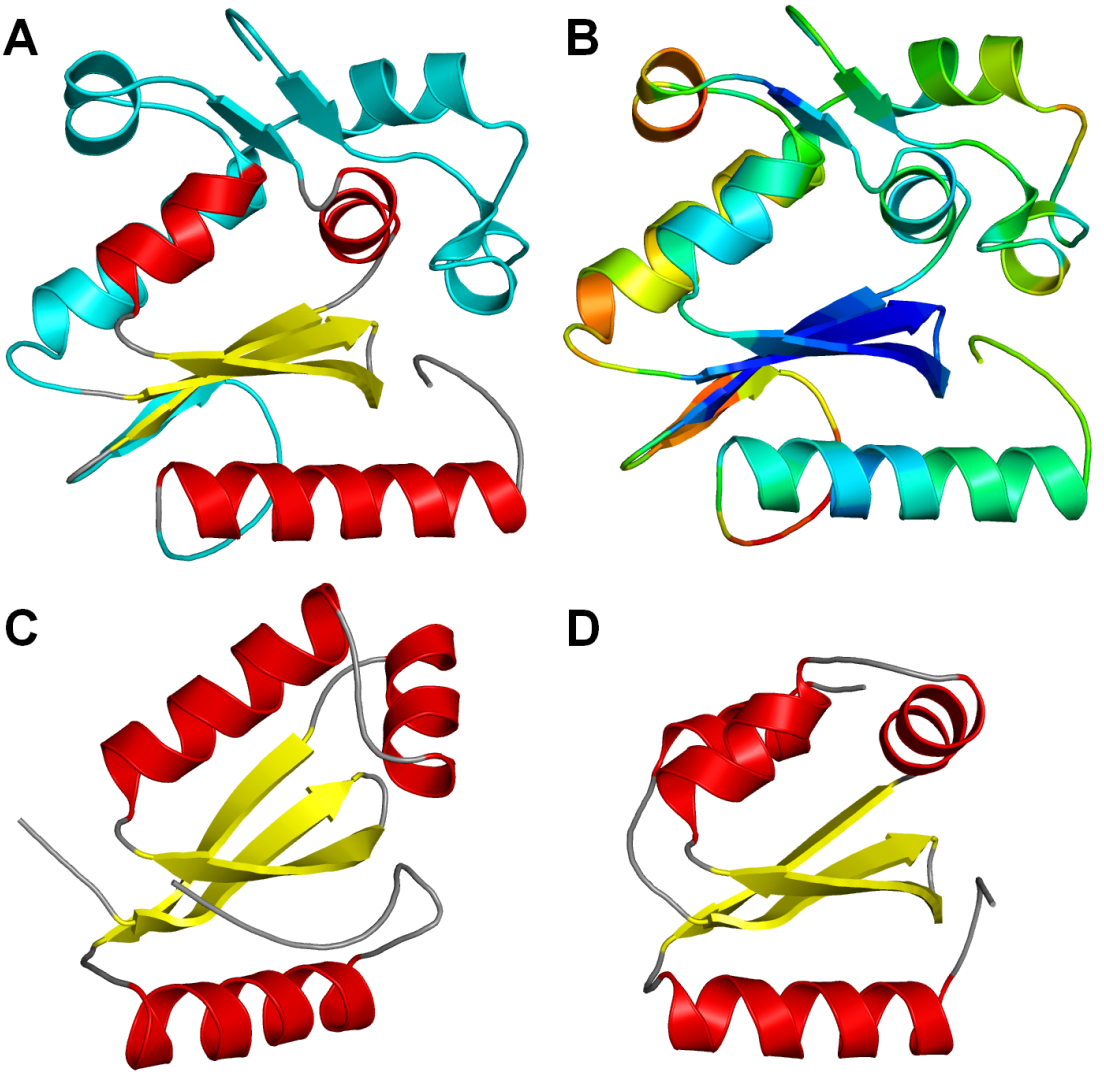
**A structural model of R.Hpy188I (A and B) compared to its experimentally characterized homologs (C and D).** Coordinates of the R.Hpy188I model are available for download from the FTP server  (A) R.Hpy188I with the homology-modeled part colored according to secondary structure (helices in red, strands in yellow, loops in grey) and regions modeled *de novo *shown in cyan. (B) R.Hpy188I model colored according to the MetaMQAP score. Reliable regions are colored blue, less reliable regions are colored green (predicted deviation from the native structure ~3 Å) unreliable regions are colored yellow to red. (C) GIY-YIG domain in I-TevI (1mk0). (D) GIY-YIG domain in UvrC (1yd0).

Initially, we attempted to fold regions 1–59 (N-terminal extension), 89–103, and 128–143 (two insertions and a structure of low sequence similarity to the template) using ROSETTA (see Methods), while keeping the rest of the model 'frozen'. However, the resulting models (low-energy representatives of the 5 largest clusters of decoys) exhibited relatively poor packing (data not shown). Thus, we subjected these models to refinement with the REFINER method [38], using additional restraints on secondary structure, according to the consensus prediction reported by the meta-server. Recently, we have used this approach to correctly predict the structures of MiaA, MiaB, and MiaE enzymes [39]. Among all the refined R.Hpy188I models, the one with the lowest predicted deviation to the native structure (root mean square deviation from the native structure of about 4.25 Å according to the MetaMQAP method, and LGscore of 3.536 i.e. 'very good model' according to PROQ) has been selected as the final model (Figure 2) and subjected to further analyses.

### Analysis of the R.Hpy188I model

**Figure 3:**
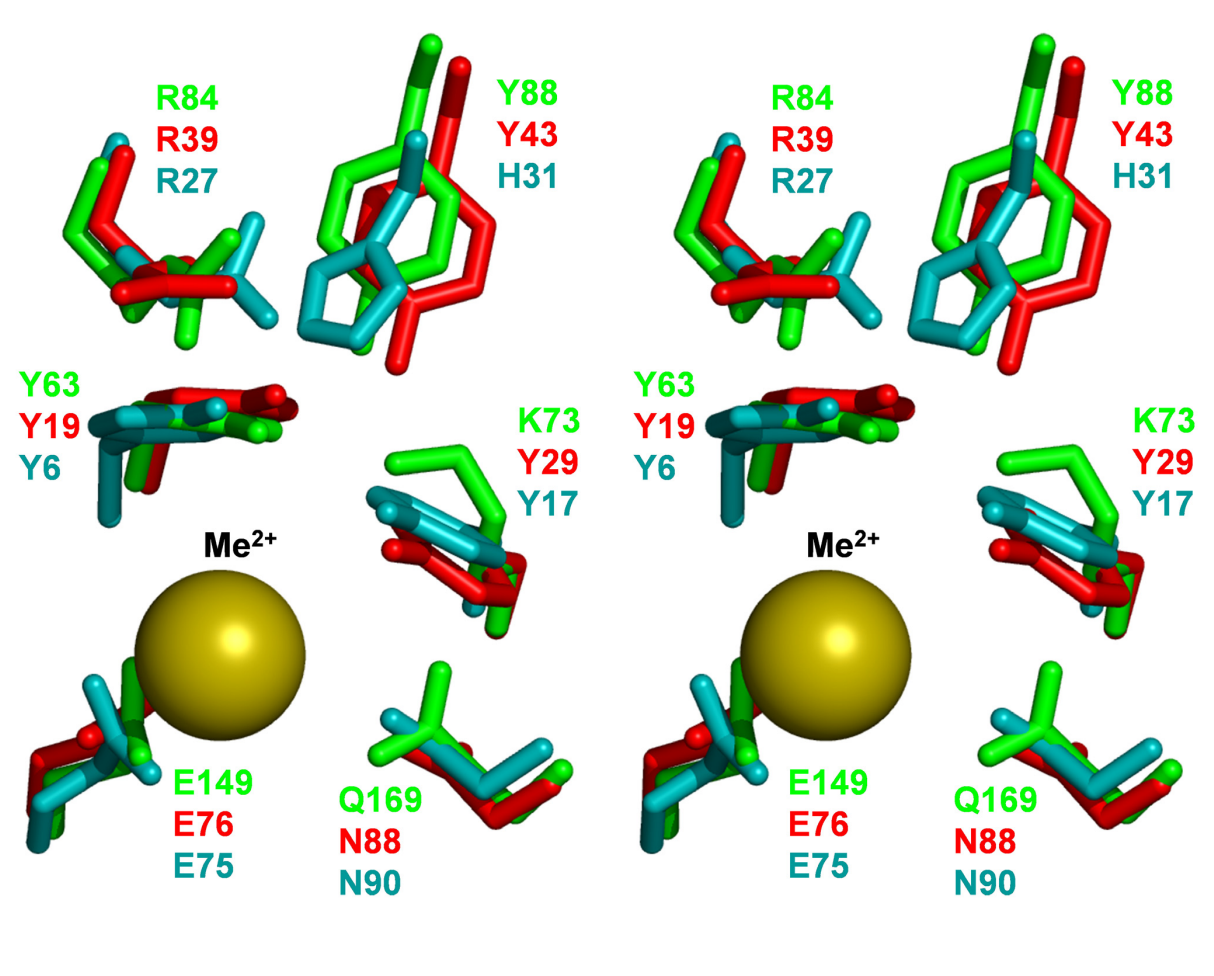
**Superposition, in stereo view, of the predicted active site residues of R.Hpy188I (green), UvrC (1yd0; blue) and I-TevI (1mk0; red).** A divalent metal ion (Mn^2+ ^in the case of the 1yd0 structure) is shown as a yellow sphere.

**Figure 4:**
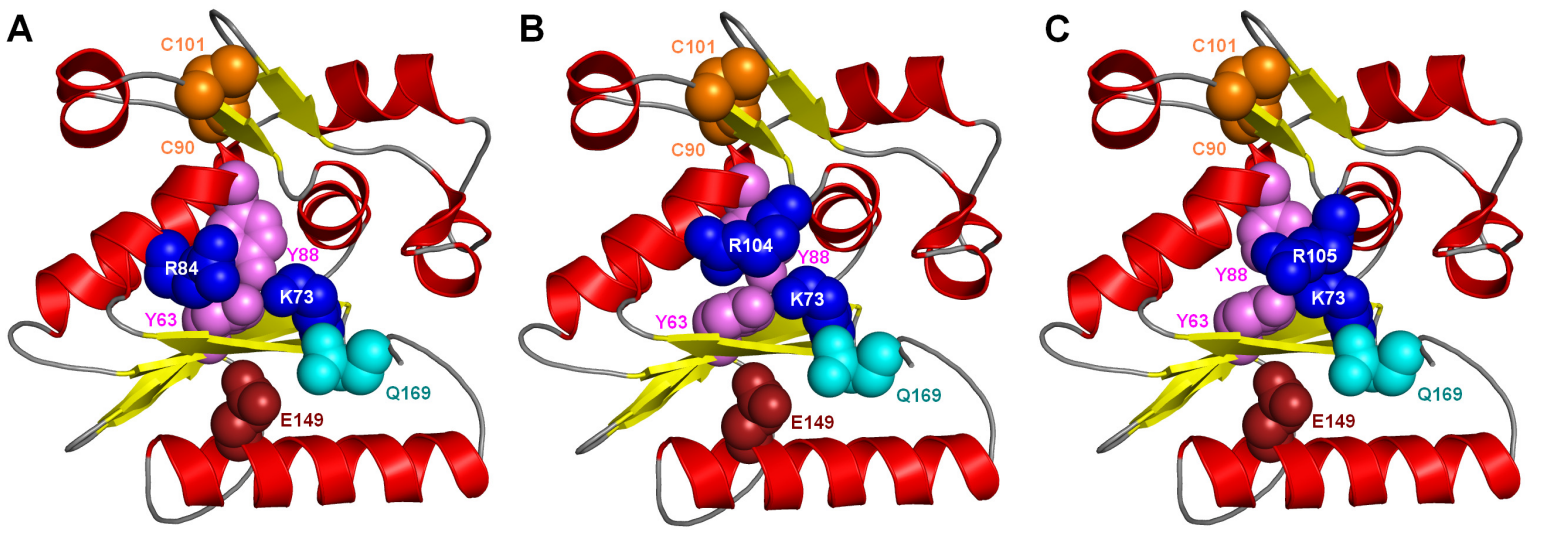
**A structural model of R.Hpy188I with presumptive active site residues and the pair of cysteines (A) compared with alternative locations of the R84 residue transferred to positions 104 or 105, i.e. "computationally modeled" double mutants R84A/T104R (B) and R84A/N105R (C), respectively.** The orientation of models is the same as in Figure 2.

Comparison of the R.Hpy188I model with the much smaller template structures of GIY-YIG domains of UvrC and I-TevI homing endonuclease (Figure 2) illustrates the challenge of modeling, in particular with respect to regions that have no counterpart in the templates and have been added *de novo*. Nonetheless, our model obtained very good scores, which suggests that it is likely to be well-folded and that potential errors are unlikely to occur in the structurally most important regions. Parts modeled *de novo *do not form an autonomously folded (sub)domain. Instead, they pack against the homology-modeled GIY-YIG core. The secondary structure in the model fulfills the restraints used during model building; interestingly, a part of the N-terminal loop (residues 6–8) has formed a small β-sheet with a part of the insertion (residues 101–103). The model reveals the predicted configuration of the putative active site of R.Hpy188I, comprising amino acid residues Y63, K73, R84, Y88, E149, and Q169 (Figure 3). In comparison with the GIY-YIG domains analyzed so far [22], R.Hpy188I and some of its homologs are the first to exhibit K (K73 for R.Hpy188I) at position corresponding to Y29 of UvrC and Y17 of I-TevI (Y of the YIG half motif) and Q (Q169 for R.Hpy188I) at the position corresponding to N88 of UvrC and N90 of I-TevI (Figure 3 and 4).

The mechanism of phosphodiester bond hydrolysis has not been elucidated experimentally for any protein from the GIY-YIG superfamily, however a tentative mechanism has been proposed based on analysis of the crystal structure of a GIY-YIG domain from the UvrC enzyme [31]. In analogy to that tentative mechanism, the divalent metal ion may function as Lewis acid, while E149 of R.Hpy188I may be responsible for metal coordination, K73 (alternatively Y63 or Y88) may function as a general base, and R84 may stabilize the negative charge of the free 5' phosphate after DNA cleavage. The hydoxyl group of Y29 of UvrC has been proposed to accept a proton from a nucleophilic water molecule while simultaneously transferring its proton to the metal-bound hydroxide [31]. The amino group of K73 might act in a similar way.

Interestingly, among the afore-mentioned residues of the putative active site, R84 (indicated by an asterisk in Figure 1) is not absolutely conserved in the R.Hpy188I family alignment. However, in a number of R.Hpy188I homologs, a corresponding Arg residue (indicated in Figure 1) is found not in the α-helix, but in another loop, on the opposite side of the active site (positions 104 or 105 in R.Hpy188I). The distributions of R84 and R104/105 are exactly complementary. Modeling of the active site variants with the Arg residue in these alternative locations (Figure 4) revealed that the positively charged guanidino group at the tip of its side chain can assume spatially similar location as in the "orthodox" position. This finding suggests that R104/105 may fulfill the same role of phosphate binding as R84 despite being attached to a non-homologous position in the protein backbone. Such a spatial "migration" of a catalytic residue has not yet been observed in enzymes from the GIY-YIG superfamily; however, it has been reported for two different residues (Glu/Asp or Lys/Arg) in a number of nucleases from the PD-(D/E)XK superfamily [40-42]. Thus, it will be interesting to test experimentally the functional significance of the swapped Arg residue in the newly discovered GIY-YIG enzymes described in this work.

In addition to potential catalytic residues, the model of R.Hpy188I (Figure 4) revealed a pair of semi-conserved cysteines (C90 and C101) in the vicinity of the alternative positions of the afore-mentioned Arg residue (84 and 104/105). The presence of these two Cys residues is strongly correlated: they co-occur in 11 sequences and a single member of this pair is present only in 2 sequences. Both Cys residues are absent from all 12 members of the R.Hpy188I family that possess a shorter variant of the intervening loop (Figure 1). It is tempting to speculate that this pair of Cys residues might have a functional role, e.g. somehow stabilize the longer variant of the loop that may be involved in protein-DNA interactions. In the model they are sufficiently close to each other to form a disulfide, which is however unlikely to happen in nature due to the generally reducing environment of the bacterial cytoplasm, which prevents oxidation of sulfhydryl groups [43]. Alternatively, if R.Hpy188I forms a dimer like most of Type II restriction endonucleases, they could form an intermolecular Zn-bindig site. Unfortunately, our model cannot provide detailed clues as to the function of C90 and C101, hence we propose them as interesting targets for experimental characterization.

**Figure 5:**
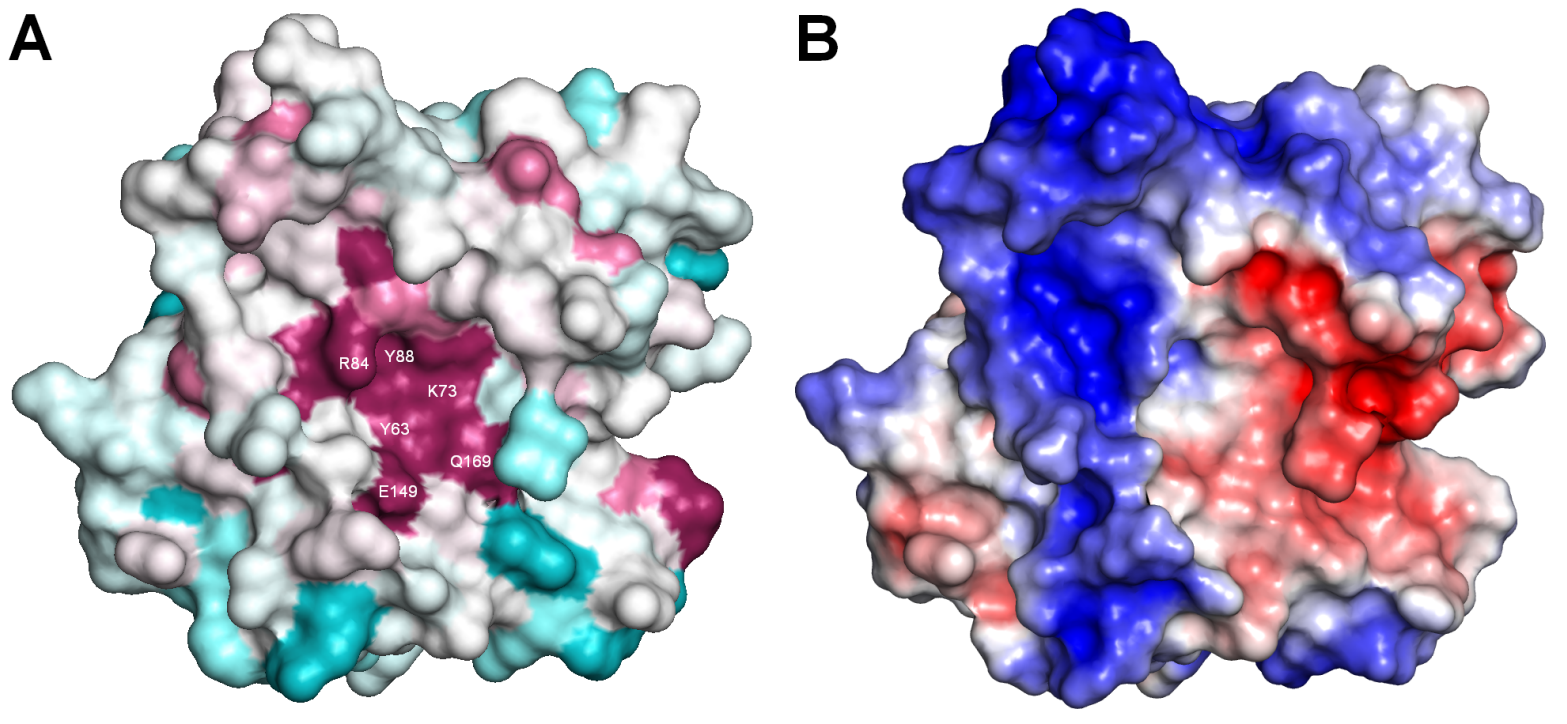
**Surface features in the R.Hpy188I model.** The orientation is the same as in Figure 2. (A) Sequence conservation: conserved regions are colored purple, variable regions are colored teal, while average regions are white. (B) Electrostatic potential: positively and negatively charged regions are colored in blue and red, respectively.

Analysis of the protein surface with respect to the distribution of sequence conservation and the electrostatic potential (Figure 5) reveals that the surface of R.Hpy188I is mostly positively charged. The predicted catalytic residues line up a bottom of a pocket with an overall neutral charge that is surrounded by a charged rim. Most of that rim exhibits positive charge (complementary to the negative charge of DNA backbone), suggesting its possible role in the DNA binding. However, one side of the rim exhibits local concentration of negative charge, suggesting potential involvement in interactions with the positively charged metal ion.

### Phylogenetics and genomic context analysis of the R.Hpy188I family

**Figure 6:**
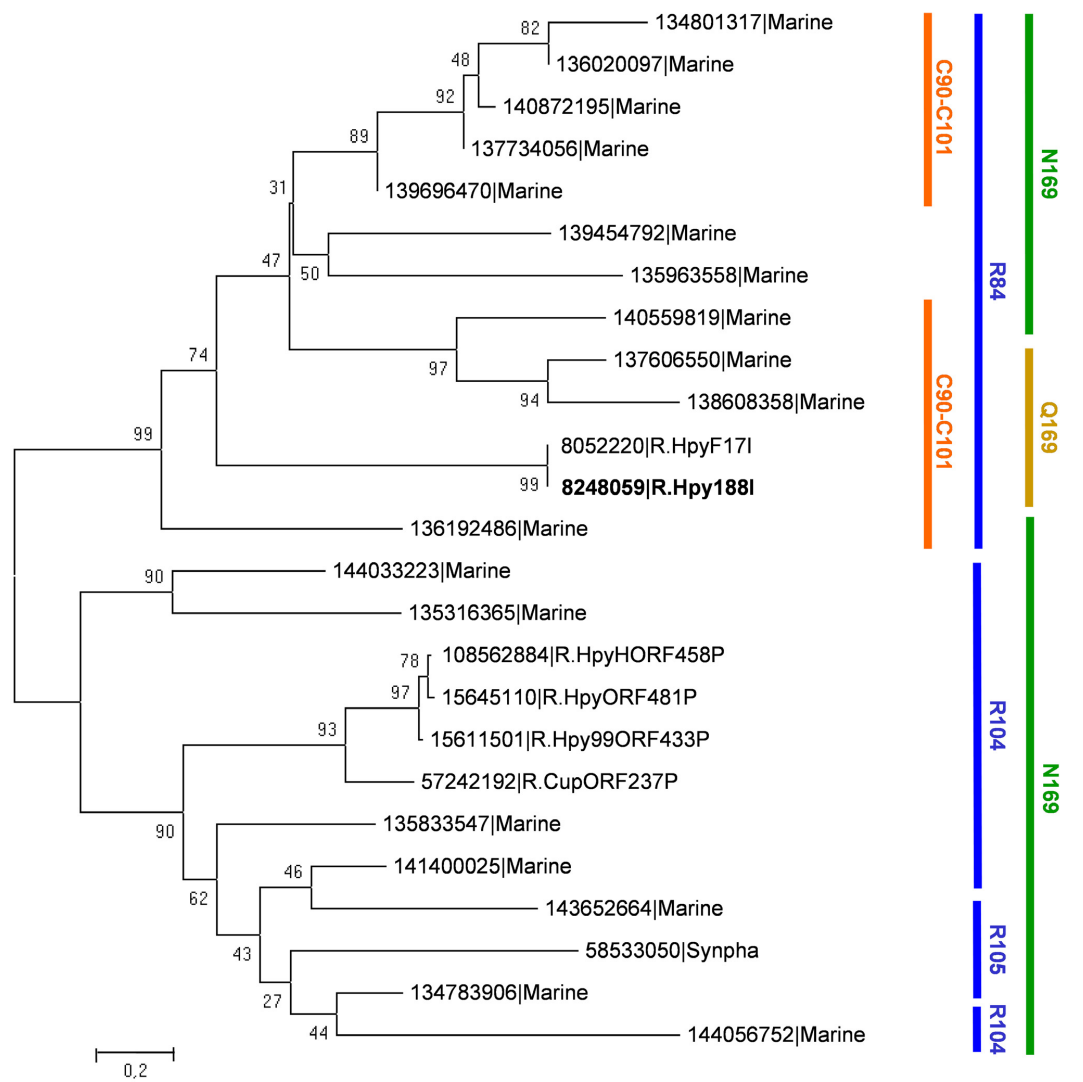
**A minimum evolution phylogenetic tree of the R.Hpy188I family.** The branches of the tree are indicated by the sequence names as in Figure 1. Values at the nodes indicate the percent value of bootstrap support. Features that distinguish different lineages are indicated on the right side of the figure (amino acid residues are numbered according to their position in the R.Hpy188I sequence).

In order to interpret the structural and genomic features of different R.Hpy188I homologs in the evolutionary context, we have calculated a phylogenetic tree for the entire family (Figure 6). It reveals that the R.Hpy188I family comprises two subfamilies with different characteristic features (hereafter dubbed R.Hpy188I branch and R.HpyAORF481P branch after the representative members from REBASE). All the members of the R.Hpy188I branch contain the phosphate-binding Arg residue at the "orthodox" 84 position. All of them, except for two sequences from environmental samples, contain the aforementioned pair of cysteines. On the other hand, members of the R.HpyAORF481P branch possess the phosphate-binding Arg in the "alternative" location (104 or 105) and lack the aforementioned pair of Cys residues (at positions corresponding to 90 and 101 in R.Hpy188I).

R.Hpy188I and its close homolog R.HpyF17I are the only members of this protein family for which some function has been determined [26]. Like virtually all Type II restriction enzymes, their genes are closely associated with genes encoding a DNA modification methyltransferase with cognate specificity. Thus, checking whether functionally uncharacterized members of the R.Hpy188I family are also genetically linked with DNA methyltransferase homologs is a convenient way to predict whether they could constitute a restriction-modification system. Unfortunately, most of R.Hpy188I members had been identified in metagenomic sequences, which typically contain only short fragments of genomic DNA and may not necessarily include the associated MTase gene together with the REase gene. Nonetheless, we carried out analysis of DNA sequence context for R.Hpy188I homologs to identify their neighbors and attempted to predict their cellular function beyond the putative generic nuclease activity.

It turned out that 19 members of the R.Hpy188I family are flanked with DNA MTase homologs (Table 1). For the remaining 6 homologs, the existence of a flanking MTase gene could not be verified because of incomplete nucleotide sequences that did not extend beyond the REase-like gene. We identified flanking MTase genes for 9 out of 13 members of the R.Hpy188I branch and 10 out of 12 of the R.Hpy481P branch (Table 1). Conserved association of members of the R.Hpy188I family with DNA MTases suggests that  all of them are or used to be a functional restriction-modification system.

**Table 1:** Genomic context analysis of R.Hpy188I family members

R.Hpy188I family member^a^		Neighboring MTase homolog			
GI (REBASE)	Truncation^b^	GI (REBASE)	Truncation^b^	Relationship to other proteins (PFAM, TIGRFAMs and BLASTP^c^)	Nucleotide sequence
R.Hpy188I branch					
8248059 (R.Hpy188I)	-	8248058 (M.Hpy188I)	-	Experimental [26]	8248057
8052220 (R.HpyF17I)	-	8052219 (M.HpyF17I)	-	TIGR02986, M.Hpy188I	8052218
137606550 (Marine)	5'	137606551	5'	M.Hpy188I	137606549 (env_nt)
138608358 (Marine)	5'	138608359, 138608360^d^	5', gap	M.Hpy188I	138608357 (env_nt)
134801317 (Marine)	5'	N.A.^e^	N.A.	N.A.	134801314 (env_nt)
136020097 (Marine)	3'	136020096	5'	M.EsaSS1928P	136020095 (env_nt)
137734056 (Marine)	5'	N.A.^e^	N.A.	N.A.	137734055 (env_nt)
139454792 (Marine)	5'	139454794, 139454795^d^	gap	M.Hpy188I	139454791 (env_nt)
139696470 (Marine)	3'	N.A.^e^	N.A.	N.A.	139696468 (env_nt)
140559819 (Marine)	5'^f ^and 3'	N.A.^e^	N.A.	N.A.	140559817 (env_nt)
140872195 (Marine)	3'^f^	140872191	5'^f^	M.EsaSS1928P	140872190 (env_nt)
135963558 (Marine)	5'	135963559, 135963560^g^	complex	M.Hpy188I	135963557 (env_nt)
136192486 (Marine)	5'	136192485, 136192487^h^	frameshift	PF02384.7, M.Hpy188I	136192484 (env_nt)
R.HpyAORF481P branch					
135833547 (Marine)	5'	135833548	3'	PF02086.6, M.HpyAORF481P	135833546 (env_nt)
144033223 (Marine)	3'	144033224	-	M.MunI	144033222 (env_nt)
141400025 (Marine)	3'	141400026	5' and 3'^f^	PF02086.6, M.HpyAORF481P	141400023 (env_nt)
143652664 (Marine)	-	143652663	-	PF02086.6, M.HpyAORF481P	143652662 (env_nt)
134783906 (Marine)	3'	N.A.^e^	N.A.	N.A.	134783905 (env_nt)
135316365 (Marine)	5'	N.A.^e^	N.A.	N.A.	135316364 (env_nt)
144056752 (Marine)	5'	144056751	3'	PF02086.6, M.HpyAORF481P	144056750 (env_nt)
108562884 (R.HpyHORF458P)	-	108562883 (M.HpyHORF458P)	-	PF02086.6, M.HpyAORF481P	108562424^i^
15611501 (R.Hpy99ORF433P)	-	15611500 (M.Hpy99ORF433P)	-	PF02086.6, M.HpyAORF481P	15611071^j^
15645110 (R.HpyAORF481P)	-	15645109 (M.HpyAORF481P)	-	PF02086.6, M.HpyAORF481P	15644634^k^
57242192 (R.CupORF237P)	-	57242195 (M.CupORF237P)	-	PF02086.6, M.HpyAORF481P	57242183^l^
58533050 (Synpha)	-	58532812 (M.SspSPM2ORFAP)	-	PF02086.6, M.HpyAORF481P	58532811

N.A., Not applicable.

^a ^The order of the sequences is the same as their order in Figure 1.

^b ^End of the cloned DNA fragment or end of the sequenced part of the cloned fragment. 5' or 3' indicate missing sequence data at the 5' or 3' side of the putative nuclease or MTase gene (corresponding to truncation of N- or C- terminus of the protein sequence, respectively).

^c ^A homologous MTase is described by its REBASE name.

^d ^The MTase gene includes an internal gap (unsequenced fragment).

^e ^A MTase gene was not detected on one side of the REase gene, but the other side is not available for analysis, thus the presence of the MTase cannot be either excluded or confirmed.

^f ^The clone contains an internal gap (unsequenced fragment), which overlaps with one end of the gene.

^g ^Probably a combination of rearrangement(s) and frameshift(s)

^h^A frameshift mutation splits the MTase gene.

^i ^Genomic neighborhood includes also M.HpyHORF460P (GI 108562885).

^j ^Genomic neighborhood includes also R.Hpy99XIP (GI 15611503) and M.Hpy99XI (GI 15611502).

^k^Genomic neighborhood includes also R.HpyAORF483P (GI 15645111) and M.HpyAORF483P (pseudogene).

^l ^Another putative RM system comprising M.CupORF235P (GI 57242193) and CUP0236 (GI 57242194; not in REBASE; a homolog of HacSORF520P from REBASE) is inserted into the CupORF237P RM system.

All but two (7 out of 9) MTases of the R.Hpy188I branch are closely related to M.Hpy188I (BLAST E-value < 1e-9). The remaining two members (GIs 136020097 and 140872195) are accompanied by truncated homologs of M.Hpy99ORF1012P and M.EsaSS1928P. In the case of 136020097 we cannot exclude that a second MTase closely related to M.Hpy188I is present on the unsequenced side of the REase gene. On the other hand, 140872195 appears to lack an M.Hpy188I homolog in its immediate neighborhood. The MTases accompanying R.Hpy188I homologs number 136020097 and 140872195 have mutually homologous sections and therefore exhibit similarity to each other. The catalytic domains of their full-length homologs M.Hpy99ORF1012P and M.EsaSS1928P are closely related to each other (33% identity), while they do not show close similarity to M.Hpy188I. This suggests that the MTase neighbors of 136020097 and 140872195 belong to a subfamily of MTases (together with M.Hpy99ORF1012P and M.EsaSS1928P) that is distinct from a subfamily of M.Hpy188I, although all these proteins belong to the same gamma class of N-MTases.

In the R.HpyAORF481P branch, 9 out of 10 detected MTase homologs are members of one family of closely related sequences, exemplified by M.HpyAORF481P. Interestingly, these proteins are members of the alpha class of N-MTases, which is topologically different from the gamma class represented by M.Hpy188I (see refs. [44,45] for reviews of classes and permutations in DNA MTases). Finally, one member of the R.HpyAORF481P branch (GI 144033223) is associated with a MTase related to M.MunI, a member of beta class of N-MTases [46]. The lack of evident sequence similarity between members of the three classes of MTases and their different topology suggests that their ancestors have diverged long before the divergence of R.Hpy188I and R.HpyAORF481P. This indicates that REases have exchanged their MTase partners at least twice in the evolution of the Hpy188I family of RM systems.

Several systems from the HpyAORF481P branch appear to be associated with another RM system (Table 1). The most interesting case is observed in the genome of *Campylobacter upsaliensis *RM3195, where another putative RM system has been inserted into the CupORF237P system comprising homologs of R.HpyAORF481P and M.HpyAORF481P. Insertion of a restriction-modification gene complex into another restriction-modification gene complex has been already suggested to have occurred in *Helicobacter pylori *[47].

## Conclusion

Our results reveal that R.Hpy188I and its homologs are new members of the GIY-YIG superfamily, despite the fact that they exhibit two deviations from the consensus catalytic motif of the superfamily. First, R.Hpy188I exhibits Lys instead of Tyr of the "YIG" half-motif. Second, in one branch of R.Hpy188I family, a presumably catalytic Arg residue is missing at its typical position in sequence, but instead is found in a non-homologous position that nonetheless allows for spatial conservation of the guanidino group potentially involved in phosphate binding. Our discovery provides important insight into sequence diversity of GIY-YIG nucleases and suggests that other members with unusual active sites might await discovery. In this context, the theoretical model of R.Hpy188I structure developed in this work will serve as a convenient guide for experimental analyses aimed at understanding of the cleavage mechanism of GIY-YIG nucleases.

Our phylogenetic analysis shows that the R.Hpy188I family can be subdivided into two branches, one comprising close homologs of R.Hpy188I itself, and the other comprising close homologs of R.HpyAORF481P. Members of either branch are characterized by a different set of features, including localization of residues predicted to participate in the enzymatic activity and possibly in structural stability. They are also found associated with MTases from different classes. Last, but not least, sequence context analyses revealed that in the family of R.Hpy188I homologs, comprising mostly sequences detected in metagenomics data, all genes that have appropriate flanking sequences present in the database, are accompanied by a putative DNA MTase gene or its fragment, suggesting that they all are or used to be functional restriction-modification systems.

## Methods

### Sequence database searches, phylogenetic analyses and genomic context analyses

Searches of the non-redundant version of current sequence database (nr) and the database of environmental protein sequences (env_nr) were carried out using PSI-BLAST [48], initially separately for nr and env_nr via the websites of NCBI and MPI-Tuebingen, and finally using the local version (against the combined nr+env_nr database). The final search was carried out with the e-value threshold of 1e-3. The multiple sequence alignment of R.Hpy188I and proteins identified in nr+env_nr database was calculated using MAFFT [49] with default parameters and refined by hand to ensure that no unwarranted gaps had been introduced within α-helices and β-strands. Finally, based on the alignment, the phylogenetic tree was calculated using MEGA 4.0 [50], employing the Minimum Evolution method with the JTT model of substitutions. The stability of individual nodes was calculated using the bootstrap test (1000 replicates) and confirmed by the interior branch test. Genomic context analyses were carried out using hmmpfam from the HMMer package [51] against the PFAM [52] and TIGRFAMs [53] databases with e-value threshold of 1e-3.

### Protein fold prediction

Preliminary structure predictions were carried out via the HHPRED server [54]. As soon as we identified (by eye) sub-optimal alignments of R.Hpy188I sequence to GIY-YIG nucleases of known structure, we resubmitted it to the GeneSilico metaserver gateway [55] for secondary structure prediction and fold recognition. Structural predictions were carried out both for the R.Hpy188I sequence alone (without success), for the alignments of R.Hpy188I with sequences from the env_nr database (with somewhat better, but still statistically insignificant results), and finally for the alignment of the sequences found by searching the combined nr and env_nr database. It is important to indicate that different FR servers use completely different scoring systems, with different scales (e.g. Z-scores, e-values, percent values etc.). Moreover, the meaning of scores changes over time and may not be the same as reported in original publications describing the methods, as servers are modified and databases grow continuously. The comparable reliability thresholds for a number of servers are estimated e.g. by the Livebench benchmark [56,57] conducted by Leszek Rychlewski and co-workers. For the servers, whose results are discussed in this work, the results of the Receiver Operator Characteristics (ROC) analysis, indicating a rough estimation of the score below which the servers' predictions become less reliable are as follows: HHSEARCH [58] probability: 0.629, FFAS [59] score: -8.9 (here the scale is inverted, i.e. lower scores are better), mGenTHREADER [60] probability: 0.351, FUGUE [61] score: 7.00, INUB [62] score: 25.35, PCONS [63,64] score: 0.6657. These thresholds correspond to the average servers' scores for their 8th incorrect predictions, which amount to 5% of incorrect predictions for all targets in the Livebench test set [65]. The scores were taken from the last Livebench run (Livebench-2008.2), with the sole exception of PCONS, which has not been included in Livebench-2008.2 and its score was taken from the CASP7 evaluation [66].

### Protein structure modeling

Homology modeling of the catalytic core was carried out using the "FRankenstein's monster" approach (see [32,33] for a detailed description). Briefly, preliminary models were built with MODELLER [67] based on alternative sequence alignments between R.Hpy188I and template structures obtained from various fold-recognition servers with significant scores (all templates used for modeling were members of the GIY-YIG superfamily). The preliminary models were scored by MetaMQAP [68] and a "hybrid" model was generated by merging fragments with consensus alignment with those non-consensus fragments that exhibited best MetaMQAP scores. Additional evaluations of protein structure quality were carried out with PROQ [69].

The "hybrid" model obtained was used as a starting point for folding simulations of the complete sequence using ROSETTA [70]. The homology-modeled core of R.Hpy188I (residues 60–88, and 104–112) was completely "frozen" and the search of conformational space for the variable regions (residues 122–170) was restricted by the choice of fragments from known crystal structures that were compatible with the sequence and predicted secondary structure of R.Hpy188I. However, the models obtained by this protocol exhibited relatively poor packing and unsatisfactory MetaMQAP and PROQ scores (data not shown). Therefore, the final simulation of R.Hpy188I folding was conducted by the REFINER method, which uses a reduced representation of the protein chain and a statistical potential of mean force to describe intramolecular interactions [38]. REFINER is a real-space version of a lattice-based algorithm CABS [71] we have earlier successfully combined with the "FRankenstein's Monster" method in CASP6 [72] or for modeling of R.Eco29kI enzyme, another member of the GIY-YIG superfamily [23]. The folding was carried out with restraints on predicted secondary structure. Models generated during the simulation had their full-atom representation rebuilt and were scored using PROQ and MetaMQAP. The best-scoring structure (in terms of predicted root mean square deviation with respect to the unknown true structure) was selected as the final model. Mapping of sequence conservation onto the model was done for the multiple sequence alignment of the Hpy188I family, initially with COLORADO3D [73], and ultimately with the ConSurf server [74].

## Authors' contributions

MK carried out initial sequence database searches, and was the first to identify the potential GIY-YIG motif in R.Hpy188I by eye. He also carried out genomic context analyses and classified homologs. KHK carried out sequence database searches, structure predictions with ROSETTA, calculated the phylogenetic tree, provided detailed description of the model, prepared the alignment and the figures. MB carried out model refinement with REFINER. IK participated in the design and coordination of this study. JMB conducted fold-recognition analysis, built the template-based model, and drafted the manuscript. All authors contributed to analysis of the data and to writing of the manuscript. All the authors have read and approved the final manuscript.
